# Carrington’s Disease: A Case Report

**DOI:** 10.7759/cureus.103473

**Published:** 2026-02-12

**Authors:** Siham Bouali, Sara Gartini, Meriem Rhazari, Afaf Thouil, Hatim Kouismi

**Affiliations:** 1 Department of Pulmonology, Mohammed VI University Hospital, Mohammed First University, Oujda, MAR; 2 Department of Respiratory Diseases, Research and Medical Sciences Laboratory, Faculty of Medicine and Pharmacy of Oujda, Mohammed VI University Hospital, Mohammed First University, Oujda, MAR; 3 Department of Respiratory and Allergic Diseases, Mohammed VI University Hospital, Mohammed First University, Oujda, MAR

**Keywords:** carrington’s disease, chronic eosinophilic pneumonia, corticosteroid therapy, diffuse infiltrative lung disease, pulmonary hypereosinophilia

## Abstract

Idiopathic chronic eosinophilic pneumonia (ICEP), also known as Carrington’s disease, is a rare eosinophilic lung disorder characterized by pulmonary infiltrates with no identifiable cause. We report the case of a 59-year-old man with no significant medical history who presented with an eight-month history of progressive exertional dyspnea and a non-productive cough. Chest imaging showed bilateral peripheral ground-glass opacities. Laboratory evaluation revealed peripheral blood eosinophilia (2,000/mm³), and bronchoalveolar lavage demonstrated eosinophilic alveolitis (30%). The patient was treated with oral corticosteroids, resulting in complete symptom resolution and marked radiological improvement.

## Introduction

Idiopathic chronic eosinophilic pneumonia (ICEP) is a rare nosological entity among diffuse infiltrative lung diseases, with an estimated prevalence between 0% and 2.5% [1]. First described by Carrington et al. in 1969, this condition is characterized by an infiltration of the pulmonary parenchyma by inflammatory cellular elements with a predominant eosinophilic component, without an identifiable cause [2].

The pathogenesis of ICEP remains poorly understood, although it is often considered a localized pulmonary manifestation of an idiopathic hypereosinophilic syndrome (HES) [3]. Diagnosis relies on a combination of clinical, radiological, and biological findings, after the exclusion of other causes of eosinophilic pneumonias.

From a clinical standpoint, ICEP is particularly important to recognize in routine practice because of its potential for misdiagnosis. Its insidious presentation and frequent overlap with asthma or other eosinophilic lung diseases may lead to diagnostic delay. Early identification is essential, as ICEP typically demonstrates a rapid and dramatic response to systemic corticosteroid therapy, making timely diagnosis both prognostically and therapeutically significant. We present a typical case of ICEP in a middle-aged patient, illustrating the diagnostic and therapeutic specificities of this entity, and provide an updated review based on recent literature data.

## Case presentation

A 59-year-old male patient with no significant past medical history, a non-smoker, without specific occupational exposures or recent medication use, presented for evaluation of grade II Modified Medical Research Council (MMRC) exertional dyspnea associated with a non-productive cough that had been progressing over eight months, and no evidence of extrapulmonary manifestations was noted at presentation. His general condition was preserved, with no reported weight loss or fever.

Upon admission, the patient was afebrile and hemodynamically and respiratorily stable (oxygen saturation 97% on room air). The clinical pleuropulmonary examination was unremarkable. Initial laboratory findings revealed peripheral blood hypereosinophilia at 2,000/mm³ (20% of the leukocyte differential), associated with a moderate inflammatory syndrome (C-reactive protein (CRP) 15 mg/L). Immunological workup (antinuclear antibodies, antineutrophil cytoplasmic antibodies (ANCA)) was negative.

An exhaustive etiological workup was performed to rule out the main secondary causes of pulmonary hypereosinophilia: parasitic and fungal serological testing (aspergillosis) returned negative; total immunoglobulin E (IgE) levels were within normal limits (Table 1).

**Table 1:** Eosinophilia workup: key laboratory findings ANCA: antineutrophil cytoplasmic antibody; IgE: immunoglobulin E

Test	Result	Reference range
Eosinophil count	2,000/mm³	0–500/mm³
C-reactive protein	15 mg/L	<5.0 mg/L
Antinuclear antibody (ANA)	Negative	<80
ANCA (p-ANCA/c-ANCA)	Negative	<20
Total IgE	50 IU/mL	0–150 IU/mL
*Aspergillus fumigatus* serology	Negative	Titer < 1/320: non-significant reaction; titer = 1/320: doubtful reaction; titer ≥ 1/640: significant reaction in favor of deep aspergillosis

**Figure 1:**
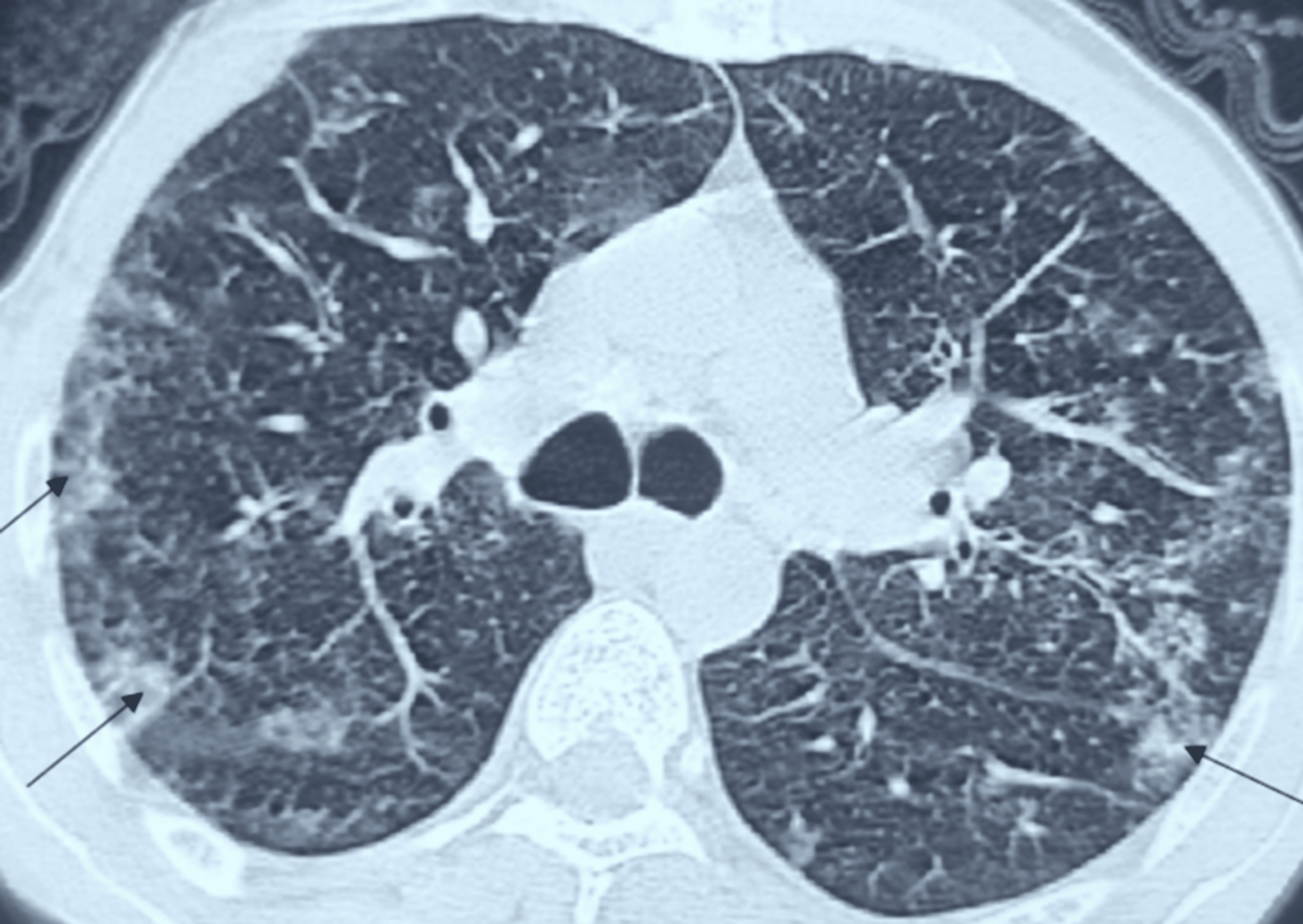
Representative axial chest CT slices showing bilateral peripheral ground-glass opacities (black arrows) CT: computed tomography

Chest computed tomography revealed bilateral peripheral ground-glass opacities, predominantly in the upper lobes (Figure 1). Pulmonary function tests were within normal limits. Bronchoalveolar lavage (BAL) demonstrated increased total cellularity with an eosinophilic predominance of 25%, confirming the diagnosis of eosinophilic alveolitis.

**Figure 2:**
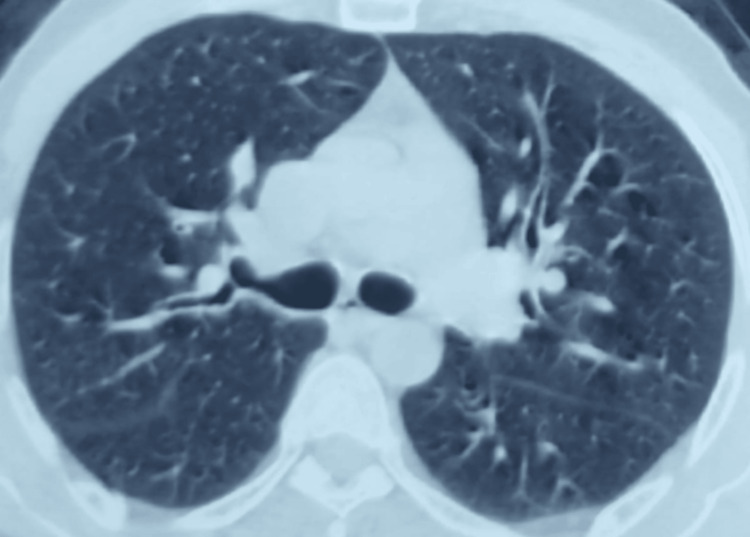
Representative axial chest CT images obtained after three months of corticosteroid therapy demonstrating near-complete radiological regression of the previously described lesions CT: computed tomography

Given this highly suggestive clinical, radiological, and biological presentation, a diagnosis of ICEP was established. Treatment with prednisolone at a dose of 1 mg/kg/day was initiated. Follow-up at three months showed complete resolution of respiratory symptoms and near-complete regression of the radiological lesions (Figure 2). A gradual tapering of the corticosteroid therapy was started, with regular clinical and radiological monitoring.

## Discussion

ICEP, historically referred to as Carrington’s disease, is considered a distinct nosological entity characterized by pulmonary infiltration with a predominant eosinophilic component and no identifiable etiology. Although it has been interpreted as a localized manifestation of idiopathic HES [3], it is distinguished by its exclusive pulmonary tropism.

ICEP predominantly affects middle-aged adults, with peak incidence observed between 40 and 50 years of age. Pediatric cases remain exceptional but have been documented in the literature [4]. The clinical presentation typically follows a subacute or chronic course, combining respiratory manifestations (persistent cough, dyspnea-often wheezy and initially suggestive of asthma) with non-specific systemic symptoms (general malaise, low-grade fever). This non-specific nature accounts for the average diagnostic delay of four to six months reported in most case series [5].

The exclusively pulmonary nature of this condition is a crucial diagnostic feature, requiring the absence of extrapulmonary involvement to rule out differential diagnoses, particularly eosinophilic granulomatosis with polyangiitis (EGPA) (formerly Churg-Strauss syndrome) and other systemic vasculitis. The differential diagnosis can be organized into primary (indeterminate-origin) disorders, such as acute and chronic eosinophilic pneumonia, as well as systemic disease-associated entities like EGPA and chronic idiopathic HES, and secondary causes (determined-origin) including parasitic infections (e.g., ascariasis, tropical pulmonary eosinophilia, and strongyloidiasis), non-parasitic infections (e.g., coccidioidomycosis and tuberculosis), aspergillosis and allergic bronchopulmonary mycoses, and drug-, toxin-, or radiation-induced eosinophilic pneumonia. In addition, several other pulmonary conditions may involve the lungs with only occasional eosinophilia, including cryptogenic organizing pneumonia, pulmonary fibrosis, pulmonary Langerhans cell histiocytosis, sarcoidosis, granulomatosis with polyangiitis, malignancy-associated eosinophilia (e.g., eosinophilic leukemia and certain lymphomas), and transplant rejection [6].

Thoracic imaging, especially computed tomography, plays a central role in diagnosis. Typical findings include ground-glass opacities or alveolar consolidations with a peripheral and migratory distribution, predominantly affecting the upper and middle lobes [7]. The labile nature of these lesions under corticosteroid treatment is a hallmark feature.

Peripheral blood eosinophilia (>500/mm³), while highly suggestive, is not constant and may be absent in approximately 10% of cases [8]. BAL represents a key examination, consistently demonstrating eosinophilic alveolitis with an eosinophil percentage typically around 25%-40% [9].

Pulmonary function tests, while not essential for diagnosis, are of clear value for follow-up, particularly in patients with coexisting asthma. At initial evaluation, spirometry may reveal an obstructive or restrictive ventilatory defect [10]. The presence of early alveolar eosinophilia is frequently associated with bronchial obstruction [11]. Arterial blood gas analysis typically shows mild hypoxemia, in contrast to acute eosinophilic pneumonia, where acute respiratory failure is more common [5].

Histological confirmation by lung biopsy is not routinely required to establish the diagnosis [12]. When performed, biopsy typically shows infiltration of the interstitium and alveolar spaces by eosinophils and lymphocytes, without evidence of vasculitis or granuloma.

A positive diagnosis rests on the combination of four major criteria [13]: (1) the presence of subacute or chronic respiratory symptoms, often accompanied by systemic manifestations; (2) evidence of peripheral blood and/or alveolar eosinophilia; (3) pulmonary opacities that are predominantly alveolar on imaging; and (4) exclusion of other causes of eosinophilic pneumonia.

First-line therapy consists of systemic corticosteroids, with initial prednisone-equivalent doses typically in the range of 0.5 to 1 mg/kg/day [14]. Clinical response is usually rapid and dramatic and may even serve as a therapeutic diagnostic test. Complete radiological improvement is often observed as early as the second week of treatment [9].

However, the long-term course is frequently characterized by relapses during tapering of therapy (up to 50% of cases), necessitating prolonged treatment beyond one year in more than two-thirds of patients [5,14]. Rare cases of fibrosing progression have been described, justifying long-term surveillance [14]. It should be noted that spontaneous remission occurs in approximately 10% of cases [9].

## Conclusions

ICEP remains a distinctive diagnostic and therapeutic entity within diffuse infiltrative lung diseases. Its diagnosis relies on a multidisciplinary approach that combines clinical features, characteristic imaging, and the presence of peripheral and/or alveolar eosinophilia. Corticosteroid therapy is the reference treatment, typically yielding an excellent initial response, but with a non-negligible risk of relapse, requiring a gradual taper and prolonged follow-up.

## References

[1] Cottin V, Cordier JF (2005). Eosinophilic pneumonias. Allergy.

[2] Carrington CB, Addington WW, Goff AM, Madoff IM, Marks A, Schwaber JR, Gaensler EA (1969). Chronic eosinophilic pneumonia. N Engl J Med.

[3] Wechsler ME, Akuthota P, Jayne D (2017). Mepolizumab or placebo for eosinophilic granulomatosis with polyangiitis. N Engl J Med.

[4] Oermann CM, Panesar KS, Langston C (2000). Pulmonary infiltrates with eosinophilia syndromes in children. J Pediatr.

[5] Jeong YJ, Kim KI, Seo IJ (2007). Eosinophilic lung diseases: a clinical, radiologic, and pathologic overview. Radiographics.

[6] Butt NM, Lambert J, Ali S (2017). Guideline for the investigation and management of eosinophilia. Br J Haematol.

[7] Johkoh T, Müller NL, Akira M (2000). Eosinophilic lung diseases: diagnostic accuracy of thin-section CT in 111 patients. Radiology.

[8] Allen JN, Davis WB (1994). Eosinophilic lung diseases. Am J Respir Crit Care Med.

[9] Pope-Harman AL, Davis WB, Allen ED, Christoforidis AJ, Allen JN (1996). Acute eosinophilic pneumonia. A summary of 15 cases and review of the literature. Medicine (Baltimore).

[10] Durieu J, Wallaert B, Tonnel AB (1997). Long-term follow-up of pulmonary function in chronic eosinophilic pneumonia. Groupe d'Etude en Pathologie Interstitielle de la Société de Pathologie Thoracique du Nord. Eur Respir J.

[11] Naughton M, Fahy J, FitzGerald MX (1993). Chronic eosinophilic pneumonia. A long-term follow-up of 12 patients. Chest.

[12] Jederlinic PJ, Sicilian L, Gaensler EA (1988). Chronic eosinophilic pneumonia. A report of 19 cases and a review of the literature. Medicine (Baltimore).

[13] Marchand E, Reynaud-Gaubert M, Lauque D, Durieu J, Tonnel AB, Cordier JF (1998). Idiopathic chronic eosinophilic pneumonia. A clinical and follow-up study of 62 cases. The Groupe d'Etudes et de Recherche sur les Maladies "Orphelines" Pulmonaires (GERM"O"P). Medicine (Baltimore).

[14] Marchand E, Cordier JF (2006). Idiopathic chronic eosinophilic pneumonia. Orphanet J Rare Dis.

